# Assessing the influence of third molar classification on the risk of caries in adjacent teeth

**DOI:** 10.55730/1300-0144.6160

**Published:** 2025-10-13

**Authors:** Sinan Yasin ERTEM, Fatih DOĞANOĞLU, Fehime Alkan AYGÖR

**Affiliations:** 1 Department of Oral and Maxillofacial Surgery, Faculty of Dentistry, Ankara Yıldırım Beyazıt University, Ankara, Turkiye; 2 Department of Restorative Dentistry, Faculty of Dentistry, Ankara Yıldırım Beyazıt University, Ankara, Turkiye

**Keywords:** Caries, third molar, impaction, angulation

## Abstract

**Background/aim:**

This study aimed to evaluate the relationship between the impaction level and angulation of third molars and the incidence of caries in the adjacent second molars.

**Materials and methods:**

A total of 690 panoramic radiographs obtained from patients at Ankara Yıldırım Beyazıt University between May 2022 and January 2024 were retrospectively analyzed. Third molars were classified based on their eruption status using the Pell and Gregory classification (levels A, B, and C), and angulation (mesioangular, distoangular, vertical, horizontal, and inverted). The presence of caries in adjacent second molars was assessed using the international caries detection and assessment system/international caries classification and management system. The association between third molar position and the presence of caries in second molars was statistically evaluated.

**Results:**

Among the 1164 third molars assessed, 52.9% were located in the mandible, and 75.2% were in a vertical position. Carious lesions were identified in 22.3% of third molars. Adjacent mandibular second molars had caries in 53.2% of cases, compared to 35.6% in the maxilla. According to the Pell and Gregory classification, class I, level A mandibular third molars were associated with the highest incidence of caries in adjacent second molars (159 out of 616). Furthermore, when only impacted teeth were considered, mesioangular impactions were associated with the highest prevalence of caries, observed in 41.1% of cases (51 out of 123).

**Conclusion:**

The findings indicate that the position and angulation of third molars play an important role in the risk of caries development in adjacent second molars. In the mandible, level A third molars in vertical (65.2%) and mesioangular (41.1%) positions had the greatest risk for caries development in adjacent second molars, whereas in the maxilla, the highest risk was found in vertical (47.3%) and mesioangular (25.7%) impactions. These results underscore the importance of early clinical evaluation and the implementation of preventive strategies to minimize caries risk in second molars associated with impacted third molars.

## 1. Introduction

Third molars are the most commonly impacted teeth in human dentition [1]. The primary etiological factors contributing to their impaction include delayed maturation and inadequate space within the dental arch. Additionally, insufficient skeletal growth, morphological and positional abnormalities of the teeth, increased regional bone density, local infections, cystic lesions, systemic diseases, and genetic syndromes can contribute to partial or complete impaction of third molars [2].

While impacted third molars may remain clinically asymptomatic, they are frequently associated with various pathological conditions such as pericoronitis, root resorption of adjacent teeth, dental caries, and the development of odontogenic cysts or tumors [3]. Among these, the development of caries on the distal surface of adjacent mandibular second molars is one of the most prevalent and clinically significant pathologies [4–6]. This is particularly prevalent when the third molar is partially erupted and has a mesioangular or horizontal orientation, resulting in close contact with the cervical region of the adjacent second molar [6].

Such positioning can hinder effective oral hygiene by promoting food retention, increasing biofilm accumulation, and impairing the natural self-cleansing mechanisms of the oral cavity, particularly due to limited salivary flow in the distal area of the second molars. Consequently, the risk of caries development in these regions is elevated [7].

Early-stage distal caries on mandibular second molars often go undetected during routine clinical examinations and are frequently diagnosed only when the lesion has progressed. In many cases, the caries exceeds the possibility of restorative management, necessitating the extraction of the affected second molar. The literature suggests that nearly one-quarter of such lesions are nonrestorable at the time of diagnosis [8]. Therefore, evaluating the impaction level and angulation of third molars, along with their potential pathological effects on adjacent teeth, is of significant clinical importance for early diagnosis and appropriate intervention.

Dental caries is a multifactorial disease arising from the complex interplay of host susceptibility, microbial presence, dietary habits, oral hygiene status, time, and salivary properties [8]. In this context, investigating the influence of third molar position—particularly impaction depth and angulation—on the development of distal caries in adjacent second molars may provide valuable insights into the mechanisms of caries pathogenesis. Such understanding may further improve preventive and prophylactic approaches in clinical dental practice. Moreover, the findings of this study may help dental practitioners anticipate the risk of caries development in second molars associated with the position of third molars. Consequently, it could facilitate the implementation of preventive treatment strategies, such as prophylactic extraction of third molars, alongside clinical evaluations to prevent caries in second molars.

In the current literature, particularly over the past 5 years, few studies that have thoroughly evaluated the association between third molar eruption classification and the incidence or severity of caries in adjacent second molars. The aim of this retrospective study was to assess whether the impaction level and angulation of mandibular third molars influence the incidence and severity of caries in adjacent second molars.

## 2. Materials and methods

This retrospective study investigated the influence of eruption status and angulation of mandibular third molars on the incidence of caries in adjacent second molars. Panoramic radiographs were collected from patients who presented to the Faculty of Dentistry at Ankara Yıldırım Beyazıt University between May 2022 and January 2024. Ethical approval was obtained from the Ankara Yıldırım Beyazıt University Health Sciences Ethics Committee (date: 21 March 2024, approval number: 2024-621) prior to data collection. Initially, 710 panoramic radiographs were reviewed; however, 2.9% were excluded due to exposure-related artifacts, resulting in 690 images included in the final analysis. When panoramic images were insufficient for assessing caries, additional periapical or bitewing radiographs were consulted to improve diagnostic accuracy.

The inclusion criteria required the presence of at least one mandibular third molar adjacent to a second molar, along with high-quality radiographs suitable for interpretation. Exclusion criteria included patients under 20 years of age, absence of the relevant molar teeth, or the presence of restorations (fillings or crowns) on the evaluated second molars. All radiographs were acquired using an orthopantomograph (Planmeca, Helsinki, Finland) under standardized imaging protocols. All evaluations were performed by a single experienced clinician to ensure consistency. Third molars were classified by eruption status (fully erupted, partially erupted, or impacted) and by impaction depth according to the Pell and Gregory classification: level A (occlusal surface at the level of the second molar), level B (crown aligned with the midroot), and level C (crown located below the root level). The status of the mandibular third molar in relation to the anterior border of the mandibular ramus was categorized into three classes. In class I, sufficient space exists between the distal surface of the second molar and the anterior border of the ramus to accommodate the mesiodistal diameter of the third molar crown. In class II, the available space is less than the mesiodistal width of the crown, and the tooth is partially embedded within the ramus. In class III, the third molar is entirely positioned within the mandibular ramus, with no space available for eruption.

Winter’s classification categorizes mandibular third molars according to their angulation in relation to the long axis of the adjacent second molar. Teeth were categorized as distoangular (>90°), vertical (61–90°), mesioangular (31–60°), horizontal (0–30°), inverted (0°), or placed into alternative classifications where appropriate. The international caries detection and assessment system/international caries classification and management system (ICDAS/ICCMS) is a standardized scoring system used to clinically and radiographically assess the severity and extent of carious lesions. The radiographic detection of caries was assessed using the ICDAS/ICCMS criteria adapted for radiographic evaluation, as previously described in the ICCMS Guide for Practitioners and Educators [9,10]. 1 Caries on radiographs were identified and categorized into four stages based on radiographic appearance, corresponding to the ICDAS/ICCMS radiographic scoring system. Codes 1–4: initial-stage (confined to enamel), moderate-stage (into dentin but less than half its thickness), advanced-stage (beyond half of the dentin, not reaching the pulp), and severe-stage caries (extending into the pulp).

Statistical analyses were performed with IBM SPSS Statistics version 25.0 (IBM Corp., Armonk, NY, USA). According to an a priori power analysis performed with G*Power (version 3.1.9.4), at least 400 panoramic radiographs were needed to obtain a statistical power of 0.95, assuming an effect size of 0.25 for the chi-square test. The statistical evaluation was conducted using the Pearson chi-square (χ^2^) test. For the second molars, the presence of caries adjacent to third molars was assessed according to the ICDAS/ICCMS classification, and statistical analyses were performed to determine whether these findings differed significantly. For the third molars, caries status was similarly evaluated using the ICDAS/ICCMS classification, and the data were analyzed to investigate whether there was a statistically significant difference in the level of caries formation compared to second molars.

## 3. Results

A total of 1164 impacted third molars were identified in the examined panoramic radiographs, comprising 548 (47%) maxillary and 616 (52.9%) mandibular third molars. The distribution of these molars based on Winter’s classification and Pell and Gregory classification, and the number of carious lesions observed in both the third molars and their adjacent second molars are presented in Table 1. Regarding angulation, 75.2% of all third molars were vertical in position, followed by 11.5% in mesioangular, 8% in distoangular, 4.8% in horizontal, and 0.1% in inverted positions. According to the Pell and Gregory classification, 70.7% of third molars were classified as level A, 8.8% as level B, and 20.3% as level C. The distribution of classifications was similar between maxillary and mandibular molars. Specifically for mandibular third molars, 32.6% were class I, 21.1% were class II, and 14.4% were class III.

There was no statistically significant difference in eruption level among third molars (p = 0.751). In terms of angulation, the vertical position was more frequent than other angulations, and this difference was statistically significant (p < 0.001). There was no statistically significant difference in distribution of mandibular third molars in relation to the ramus (p = 0.745). According to the ICDAS/ICCMS classification, there was no statistically significant difference in caries development within the third molars (p = 0.239). However, a statistically significant difference was observed in caries development in the second molars (p = 0.024). Of the total 1164 third molars examined, 22.3% were carious. The severity of these carious lesions, as determined by the ICDAS/ICCMS classification, was as follows: 35.3% were stage 1, 21.9% stage 2, 20.7% stage 3, and 21.9% stage 4. Regarding adjacent second molars, a total of 178 carious lesions were identified among 505 maxillary second molars, with 68 lesions classified as ICDAS/ICCMS stage 4. In the mandible, 553 second molars were examined, of which 293 were carious, including 83 lesions categorized as stage 4. These data indicate that mandibular third molars are associated with a higher incidence and severity of caries in adjacent second molars compared to maxillary third molars. Notably, third molars categorized as class I and level A had a higher propensity for developing caries both in the third molar itself and in the adjacent second molar. Among 200 mandibular third molars classified as class I and level A, 60 were diagnosed with caries. Similarly, 51 out of 174 right mandibular third molars in the same classification had carious lesions. Additionally, 75 carious lesions were identified in the second molars adjacent to these mandibular third molars. Table 2 shows the distribution of mandibular third molars and their neighboring second molars based on angulation and the presence of caries. Table 3 shows the corresponding distribution for maxillary third molars and adjacent second molars.

After vertical positioning, mesioangular was the next most prevalent angulation. In the mandible, 98 mesioangular third molars were identified, of which 7 were carious, and 46 adjacent second molars had caries. In the maxilla, 20 mesioangular third molars were recorded; none were carious, although 5 adjacent second molars showed caries. Overall, nonvertical angulations were associated with a considerably lower prevalence of caries in both third molars and their adjacent second molars. These findings emphasize the significant influence of third molar position and angulation on the risk of caries in adjacent teeth. Consequently, these results highlight the importance of considering impaction characteristics during clinical evaluation and treatment planning of third molars, particularly to guide preventive strategies and reduce the progression of caries in second molars. Mesioangular and class I–level A third molars had a higher risk of distal caries in adjacent second molars, indicating the need for closer radiographic monitoring and preventive measures in patients with these impaction patterns.

## 4. Discussion

In this study, eruption status and positioning of third molars were shown to influence the occurrence of caries in adjacent second molars through the evaluation of panoramic radiographs. Studies examining the effect of third molars on caries development in second molars are relatively limited [11,12]. To the best of our knowledge, the only study focusing on this specific issue within the last 5 years was conducted by Yıldırım et al. [6], who analyzed 7998 panoramic radiographs and found the highest incidence of caries in neighboring second molars when maxillary third molars were positioned at level A. Similarly, Oderinu et al. [13] reported that mandibular third molars in a semierupted mesioangular position had the greatest potential to induce caries in adjacent second molars. Additionally, several studies have found an association between the vertical positioning of mandibular third molars and caries formation in second molars [14–16]. Most investigations evaluating the influence of third molars on caries formation have typically concentrated on unerupted or semierupted third molars [17,18]. For example, studies including only impacted and semierupted third molars have consistently concluded that semierupted, mesioangular mandibular third molars pose the highest risk for the development of caries in adjacent second molars.

Allen et al. [19] reported that 42% of second molars adjacent to partially or fully impacted mandibular third molars had caries, highlighting the pathological risk associated with these impactions. Similarly, Ye et al. [8] noted that semiimpacted third molars were associated with 20–40% higher rates of pathology. The anatomical position and posterior location of third molars in the dental arch make them inherently less accessible for mechanical cleaning, contributing to their increased susceptibility to caries. This study showed that vertically positioned third molars had the highest rate of caries formation at contact points with adjacent second molars (70.7%). These third molars create regions that are particularly challenging to clean, facilitating the accumulation of bacterial plaque and food debris. Interproximal contact areas between third and second molars also contribute to caries development, which are especially difficult to clean [20]. Although the occlusal and other surfaces may undergo partial cleaning during masticatory activity, these contact points often escape adequate hygiene due to their posterior location and anatomical limitations. In the current study, vertical third molars were most frequently associated with caries development in adjacent second molars, followed by mesioangular third molars, which accounted for 11.5% of cases. After excluding vertically oriented third molars from the analysis, 46.5% (134/288) of the remaining third molars had mesioangular angulation, with 41.1% (51/123) contributing to caries in the neighboring second molars. These findings are consistent with previously published studies emphasizing the caries-inducing potential of mesioangular third molars. Nevertheless, considering that vertically positioned third molars were also included in the analysis and had a significantly higher incidence of associated caries, the overall highest rate of second molar caries was attributed to third molars in the vertical position.

In this study, the distribution of third molars by jaw was examined. There was a significantly higher prevalence in the mandible (52.9%) compared to the maxilla (47.1%), which aligns with findings reported by Kumar et al. [21]. Moreover, vertical orientation was the most prevalent angulation pattern in both jaws, consistent with the literature [21,22]. However, other studies have suggested that mesioangular impaction is the most common angulation pattern [14,23]. In a recent systematic review and metaanalysis, the total prevalence of distal surface caries in second molars adjacent to impacted mandibular third molars were found to be 29.89%; the mesioangular position was associated with the highest risk (43.37%), and level A impaction was most commonly associated with caries [24]. In evaluating the potential for caries development in second molars adjacent to third molars, caries were identified in 178 of 510 maxillary second molars, corresponding to a prevalence of 34.9%. In the mandible, 293 of 553 second molars were affected (52.9%), a notably higher rate that aligns with previous reports highlighting the increased susceptibility of mandibular second molars to caries in the presence of third molars [21]. These results further support previous reports that mandibular third molars are more frequently associated with second molar caries. When the prevalence of caries in the third molars themselves was assessed, 125 out of 548 maxillary third molars (22.8%) and 135 out of 616 mandibular third molars (21.9%) had carious lesions, indicating similar susceptibility between the 2 jaws.

When caries severity in second molars was evaluated according to the ICDAS/ICCMS, a linear distribution was observed from ICDAS/ICCMS stages 1 to 4. The most frequently observed caries level was ICDAS/ICCMS stage 1 (11.4%), followed by stage 2 (10.9%), stage 3 (7.8%), and stage 4 (7.1%). These findings suggest that caries in second molars associated with third molars are generally diagnosed at early stages, with fewer cases presenting as advanced lesions. Based on the Pell and Gregory classification, level A was the most frequently observed eruption level in both the maxilla (70.4%) and mandible (71.2%). However, previous studies by Quek et al. [25] and Hassan et al. [26] reported level B as the most common impaction level, although those studies did not differentiate between fully impacted and partially erupted third molars. Our findings align with Alsaegh et al. [27], who reported level A as the most prevalent, followed by level C. Similarly, Yilmaz et al. [16] and Mahdey et al. [28] found level C to be the most frequently observed impaction level in Turkish and Malaysian populations, respectively. According to the Pell and Gregory classification, third molars in class I and class II positions were more frequently associated with second molar caries in level A and level B combinations compared to those in class III and level C. Of the 293 observed second molar caries, 69 (23.5%) were attributed to third molars in class III and level C positions, while 224 (76.5%) were linked to class I and II third molars in level A and B positions. This finding is supported by studies reporting a reduced caries risk in cases where third molars are deeply impacted and not in direct contact with adjacent teeth [20]. However, in a study conducted by Arandi et al. [29], among the ramus depth categories, class I–level C had the highest prevalence of carious mandibular second molars (29.09%), followed by class I–level B (26.87%), class II–level B (25.57%), and class I–level A (25.0%). Lower prevalence rates were observed in class II–level C (14.38%), class II–level A (13.77%), and class III–level A (11.1%), while the lowest rates were reported in class III–level B (7.14%) and class III–level C (2.89%). The lower caries rates in these cases are likely due to their lack of exposure to the oral environment and absence of plaque-retentive interproximal contact points.

While the findings of this study generally corroborate previously published research, several limitations must be considered. First, the exclusive use of panoramic radiography, which provides limited anatomical detail, may have led to an underestimation of certain findings. Second, the retrospective design precluded the collection of detailed data on participants’ oral hygiene habits and adherence to routine dental checkups, thereby limiting the ability to control for potential confounding variables. Third, the study design did not allow for comprehensive clinical assessments, which could have enriched the diagnostic accuracy and interpretation of radiographic findings. Nevertheless, to our knowledge, no study in the past 5 years has comprehensively assessed the relationship between third molar classification and the incidence of caries in adjacent second molars, including caries grading based on ICDAS/ICCMS. Future prospective studies involving larger and more diverse populations are needed to validate these findings and provide a more nuanced understanding of the topic.

The current study provides important insights into the relationship between third molar positioning and the development of caries in adjacent teeth. Even in the absence of symptoms, it may be beneficial to inform patients of the potential caries risks associated with third molars during routine clinical assessments. Education on proper oral hygiene practices, improvements in brushing techniques, and the implementation of patient-specific preventive strategies are critical for reducing caries risk. Furthermore, motivational interventions to encourage regular dental checkups in high-risk individuals may positively impact long-term oral health outcomes.

This study identified third molars in class I, level A positions associated with a higher risk of caries, both in the third molars themselves and in adjacent second molars. Additionally, mesioangular third molars in level A positions were most frequently implicated in second molar caries. These findings highlight that identification of class I, level A, mesioangular impactions may guide clinicians toward closer monitoring of these teeth or consideration of earlier prophylactic removal. Future studies that use prospective designs and including broader populations will enhance our understanding of the interplay between third molar angulation, eruption status, and caries formation, and help develop more effective preventive and clinical strategies.

## Figures and Tables

**Table 1:** A summary of the distribution of third molars with respect to position, angulation, and ramus relationship. The caries levels in adjacent second molars and in the third molars themselves are also reported.

	*Position (p=0.751)*	*Angulation (p<0.001)*	*Ramus relationship (p=0.745)*	*Caries Level on third molar ICDA/ICCMS classification (n) (p=0.239)*	*Caries level on second molar ICDAS/ICCMS classification (n) (p=0.024)*
***Right mandibular third molar***	*Level A (227)*	*Vertical (220)*	*Class I (201)*	*I (30)*	*I (37)*
	*Level B (27)*	*Mesioangular (51)*	*Clas II (65)*	*II (14)*	*II (39)*
	*Level C (56)*	*Distoangular (16)*	*Class III (44)*	*III (13)*	*III (23)*
	*None (416)*	*Horizontal (22)*		*IV (10)*	*IV (18)*
		*Inverted (0)*			*None (142)*
***Left Mandibular Third Molar***	*Level A (212)*	*Vertical (209)*	*Class I (195)*	*I (25)*	*I (42)*
	*Level B (28)*	*Mesioangular (47)*	*Class II (65)*	*II (20)*	*II (37)*
	*Level C (66)*	*Distoangular (18)*	*Class III (45)*	*III (12)*	*III (32)*
	*None (384)*	*Horizontal (34)*		*IV (11)*	*IV (23)*
		*Inverted (2)*			*None (118)*
***Right maxillary third molar***	*Level A (193)*	*Vertical (226)*		*I (15)*	*I (20)*
	*Level B (21)*	*Mesioangular (14)*		*II (10)*	*II (22)*
	*Level C (60)*	*Distoangular (29)*		*III (15)*	*III (11)*
	*None (410)*	*Horizontal (0)*		*IV (19)*	*IV (16)*
		*Inverted (0)*			*None (172)*
***Left maxillary third molar***	*Level A (192)*	*Vertical (221)*		*I (22)*	*I (22)*
	*Level B (27)*	*Mesioangular (22)*		*II (13)*	*II (18)*
	*Level C (55)*	*Distoangular (31)*		*III (14)*	*III (17)*
	*None (416)*	*Horizontal (0)*		*IV (17)*	*IV (18)*
		*Inverted (0)*			*None (155)*

**Table 2:** The distribution of mandibular third molars according to angulation classification and caries levels.

*Angulation*	*Class I*		*Class II*		*Class III*	
	*Caries on third molar*	*Caries on second molar*	*Caries on third molar*	*Caries on second molar*	*Caries on third molar*	*Caries on second molar*
*V*	*59*	*85*	*2*	*9*	*0*	*0*
*H*	*0*	*1*	*0*	*1*	*0*	*1*
*M*	*3*	*7*	*0*	*1*	*0*	*0*
*D*	*0*	*0*	*0*	*3*	*0*	*0*
*I*	*0*	*0*	*0*	*0*	*0*	*0*
	***Left mandibular third molar level B***					
*Angulation*	***Class I***		***Class II***		***Class III***	
	***Caries on third molar***	***Caries on second molar***	***Caries on third molar***	***Caries on second molar***	***Caries on third molar***	***Caries on second molar***
*V*	*1*	*2*	*0*	*1*	*0*	*0*
*H*	*0*	*0*	*0*	*1*	*0*	*2*
*M*	*0*	*1*	*0*	*2*	*0*	*1*
*D*	*0*	*0*	*0*	*0*	*0*	*0*
*I*	*0*	*0*	*0*	*0*	*0*	*0*
	***Left mandibular third molar Level C***					
*Angulation*	***Class I***		***Class II***		***Class III***	
	***Caries on third molar***	***Caries on second molar***	***Caries on third molar***	***Caries on second molar***	***Caries on third molar***	***Caries on second molar***
*V*	*0*	*1*	*0*	*4*	*0*	*2*
*H*	*0*	*0*	*0*	*6*	*0*	*6*
*M*	*0*	*2*	*0*	*8*	*0*	*4*
*D*	*0*	*0*	*0*	*0*	*0*	*1*
*I*	*0*	*0*	*0*	*0*	*0*	*0*

*Left mandibular third molar level A*						
	*Right Mandibular Third Molar Level A*					
*Angulation*	*Class I*		*Class II*		*Class III*	
	*Caries on third molar*	*Caries on second molar*	*Caries on third molar*	*Caries on second molar*	*Caries on third molar*	*Caries on second molar*
*V*	*47*	*74*	*5*	*15*	*0*	*0*
*H*	*0*	*0*	*0*	*2*	*0*	*0*
*M*	*4*	*3*	*0*	*0*	*0*	*0*
*D*	*0*	*0*	*0*	*3*	*0*	*2*
*I*	*0*	*0*	*0*	*0*	*0*	*0*
	***Right mandibular third molar level B***					
*Angulation*	***Class I***		***Class II***		***Class III***	
	***Caries on third molar***	***Caries on second molar***	***Caries on third molar***	***Caries on second molar***	***Caries on third molar***	***Caries on second molar***
*V*	*0*	*1*	*0*	*0*	*0*	*1*
*H*	*0*	*0*	*0*	*2*	*0*	*2*
*M*	*0*	*0*	*0*	*0*	*0*	*1*
*D*	*0*	*0*	*0*	*0*	*0*	*0*
*I*	*0*	*0*	*0*	*0*	*0*	*0*
	***Right mandibular third molar level C***					
*Angulation*	***Class I***		***Class II***		***Class III***	
	***Caries on third molar***	***Caries on second molar***	***Caries on third molar***	***Caries on second molar***	***Caries on third molar***	***Caries on second molar***
*V*	*0*	*0*	*0*	*1*	*0*	*3*
*H*	*0*	*0*	*0*	*1*	*0*	*3*
*M*	*0*	*0*	*0*	*8*	*0*	*8*
*D*	*0*	*0*	*0*	*0*	*0*	*1*
*I*	*0*	*0*	*0*	*0*	*0*	*0*

**Table 3:** The distribution of maxillary third molars according to angulation classification and caries levels.

*Angulation*	*Right maxillary third molar level A*		*Angulation*	*Left maxillary third molar level A*	
	*Caries on third molar*	*Caries on second molar*		*Caries on third molar*	*Caries on second molar*
*V*	*47*	*64*	*V*	*62*	*73*
*H*	*0*	*0*	*H*	*0*	*0*
*M*	*0*	*0*	*M*	*0*	*1*
*D*	*0*	*0*	*D*	*0*	*2*
*I*	*0*	*0*	*I*	*0*	*0*
***Angulation***	***Right maxillary third molar level B***		***Angulation***	***Left maxillary third molar level B***	
	***Caries on third molar***	***Caries on second molar***		***Caries on third molar***	***Caries on second molar***
*V*	*0*	*0*	*V*	*10*	*1*
*H*	*0*	*0*	*H*	*0*	*0*
*M*	*0*	*0*	*M*	*0*	*0*
*D*	*0*	*1*	*D*	*1*	*1*
*I*	*0*	*0*	*I*	*0*	*0*
***Angulation***	***Right maxillary third molar level C***		***Angulation***	***Left maxillary third molar level C***	
	***Caries on third molar***	***Caries on second molar***		***Caries on third molar***	***Caries on second molar***
*V*	*0*	*8*	*V*	*0*	*6*
*H*	*0*	*0*	*H*	*0*	*0*
*M*	*0*	*0*	*M*	*0*	*4*
*D*	*0*	*9*	*D*	*0*	*5*
*I*	*0*	*0*	*I*	*0*	*0*

## Footnotes

1 Pitts NB, Ekstrand KR. ICCMS™ guide for practitioners and educators (2014). ICDAS Foundation. Website https://www.iccms-web.com [accessed 25 September 2025]
